# Structural and Temporal Dynamics of Mesenchymal Stem Cells in Liver Diseases From 2001 to 2021: A Bibliometric Analysis

**DOI:** 10.3389/fimmu.2022.859972

**Published:** 2022-05-19

**Authors:** Bo Shao, Ya-fei Qin, Shao-hua Ren, Qiu-feng Peng, Hong Qin, Zhao-bo Wang, Hong-da Wang, Guang-ming Li, Yang-lin Zhu, Cheng-lu Sun, Jing-yi Zhang, Xiang Li, Hao Wang

**Affiliations:** ^1^ Department of General Surgery, Tianjin Medical University General Hospital, Tianjin, China; ^2^ Tianjin General Surgery Institute, Tianjin Medical University General Hospital, Tianjin, China; ^3^ Department of Respiratory and Critical Care Medicine, Tianjin Fourth Central Hospital, Tianjin, China; ^4^ School of Basic Medical Sciences, Tianjin Medical University, Tianjin, China

**Keywords:** mesenchymal stem cells, liver diseases, bibliometric analysis, visualization, citespace, VOSviewer

## Abstract

**Background:**

Mesenchymal stem cells (MSCs) have important research value and broad application prospects in liver diseases. This study aims to comprehensively review the cooperation and influence of countries, institutions, authors, and journals in the field of MSCs in liver diseases from the perspective of bibliometrics, evaluate the clustering evolution of knowledge structure, and discover hot trends and emerging topics.

**Methods:**

The articles and reviews related to MSCs in liver diseases were retrieved from the Web of Science Core Collection using Topic Search. A bibliometric study was performed using CiteSpace and VOSviewer.

**Results:**

A total of 3404 articles and reviews were included over the period 2001-2021. The number of articles regarding MSCs in liver diseases showed an increasing trend. These publications mainly come from 3251 institutions in 113 countries led by China and the USA. Li L published the most papers among the publications, while Pittenger MF had the most co-citations. Analysis of the most productive journals shows that most are specialized in medical research, experimental medicine and cell biology, and cell & tissue engineering. The macroscopical sketch and micro-representation of the whole knowledge field are realized through co-citation analysis. Liver scaffold, MSC therapy, extracellular vesicle, and others are current and developing areas of the study. The keywords “machine perfusion”, “liver transplantation”, and “microRNAs” also may be the focus of new trends and future research.

**Conclusions:**

In this study, bibliometrics and visual methods were used to review the research of MSCs in liver diseases comprehensively. This paper will help scholars better understand the dynamic evolution of the application of MSCs in liver diseases and point out the direction for future research.

## Introduction

Stem cell therapy is considered a new treatment for a cluster of diseases. Mesenchymal stem cells (MSCs), which do not display associated hematological markers, have been proven safe and effective in clinical practice for over ten years (1). MSCs are notable for lacking the expression of CD40, CD40 ligand (CD40L), CD80, CD86, and major histocompatibility class II (MHC- II), all of which are required to activate effector T cells (2). As its low immunogenicity and immunoregulatory effects, MSCs can educate immune cells and ultimately regulate the disease-specific microenvironment through direct or indirect cell-to-cell communication, such as various surface molecules and extracellular vesicles (3). MSCs have important research value and broad application prospects based on these unique properties.

Liver disease is a leading cause of global death and morbidity (4–6). Many inflammatory liver diseases do not respond to treatment, and these individuals frequently progress to end-stage liver diseases, necessitating liver transplantation. MSCs have made great progress in liver basic and clinical research in recent years. MSCs have been reported to be capable of causing the differentiation of hepatocyte-like cells, which may hold promise in augmenting liver regeneration (7). Aurich H found that under conditions favoring hepatocyte differentiation, human adipose tissue-derived mesenchymal stem cells (hAT-MSCs) obtained functions of hepatocyte differentiation, including urea production, glycogen synthesis, cytochrome P450 enzyme activity, and hepatocyte-specific transcription expression-carbamoyl phosphate synthase, albumin, and cytochrome P45 type 3A4 (CYP3A4) (8). MSCs have a hepatoprotective impact in mice with hepatic ischemia/reperfusion (I/R) damage, reducing hepatocellular apoptosis and mtROS accumulation through upregulating the PINK1-dependent mitophagy pathway (9). MSCs release the major soluble mediator milk fat globule-EGF factor 8 (MFGE8), which lowers extracellular matrix (ECM) proteins and liver fibrosis in mice by inhibiting transforming growth factor beta 1 (TGFB1)-mediated activation of hepatic stellate cells (HSCs) (10). In addition, He Y found that human umbilical cord-derived mesenchymal stem cells (hUC-MSCs) can inhibit inflammatory cell infiltration and hepatocyte apoptosis, most likely *via* inhibiting of Notch, IFN-γ/Stat1, and IL-6/Stat3 signaling in liver tissues of ACLF rats (11). These promising preclinical findings have sparked a slew of clinical investigations on MSCs. There is, however, no complete and impartial assessment on the trend of publishing outputs, influential countries/regions, institutions, or authors, and their collaboration, knowledge base, hotspots, and frontiers in MSCs and liver disease research.

The bibliometric analysis focuses on the system and features of research literature and has been widely used in qualitative and quantitative analysis of scientific literature to comprehend the knowledge structure and the relationships and clustering of studies (12, 13). Contributions from different nations, organizations, experts, and publications can be compared to describe and project future advancements in a certain study topic (14). Many scholars have used bibliometrics analysis in various fields of medicine, such as psychological disorders (15), cardiovascular disease (16), endocrine disease (17), digestive system neoplasms (18), ferroptosis (13), and biological signaling molecule (19). It is becoming increasingly important in assessing hotspots frontiers and formulating guidelines between MSCs and liver disease research.

Thus, this research explores the hotspots and frontiers trends of MSC research in liver disease over the past 20 years and forms a corresponding knowledge map with CiteSpace and VOSviewer. This study provides the latest progress, evolution path, frontier research hotspots, and future research trends in MSCs in liver diseases for basic research and clinical prevention and treatment.

## Materials and Methods

### Data Source and Retrieval Strategy

**Figure 1:**
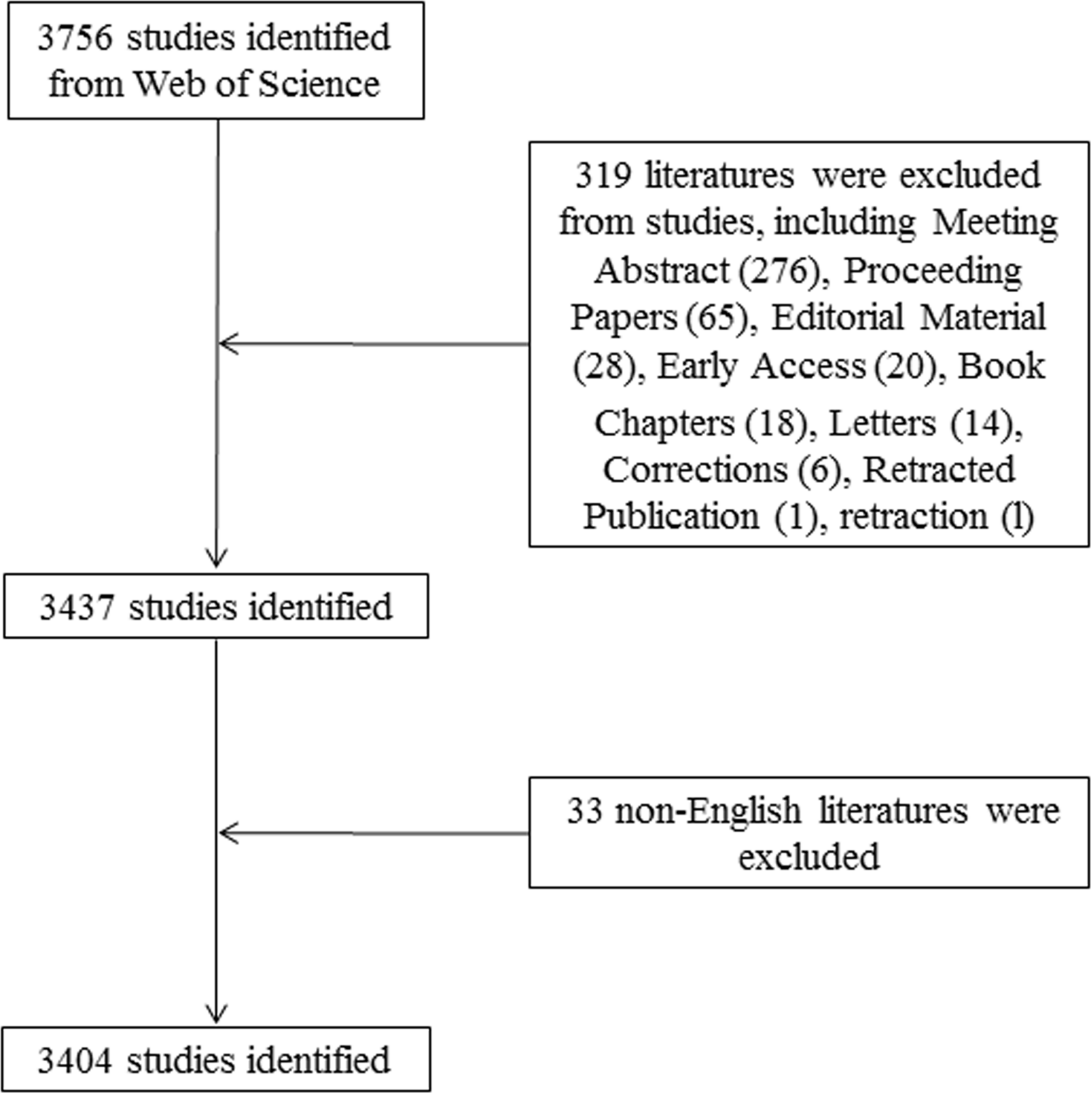
Flowchart of the screening process.

The Science Citation Index Expanded (SCI-Expanded) of the Web of Science Core Collection (WoSCC) bibliographic database developed by Thomson Scientific has been used to perform scientometric analysis (20). Considered the most influential database, WoSCC provides the most comprehensive information on bibliometric software requirements (21). The retrieval strategy used in this study was set to TS = (“hepatic” OR “liver” OR “hepatopathy”) AND TS = (“mesenchymal stem cell*” OR “mesenchymal stromal cell*”), and document retrieval is conducted in one day (October 02, 2021) to avoid deviation error. 3756 articles were retrieved, eight types of documents among them. **Table 1** shows that there were 2687 articles, accounting for 71.539% of all papers, making articles the most prevalent category of literature. There were 744 reviews in second place, accounting for 19.808% of the total. The other 8 document types were meeting abstracts (276), proceedings papers (65), editorial materials (28), early access (20), book chapters (18), letters (14), corrections (6), retracted publications (1) and retractions (1). Two researchers (BS and YFQ) independently searched the original data, then discussed possible differences, and the final agreement reached 0.95 (22), showing substantial consistency. We ultimately analyzed 3404 articles, and the detailed filtering process is shown in **Figure 1**.

**Table 1:** Document types of the publications.

Rank	Document Types	TP	% of 3,756	CAWS	TCAWS	h-index
1	Articles	2,687	71.539	53,504	86,354	131
2	Review Articles	744	19.808	27,948	31814	82
3	Meeting Abstracts	276	7.348	32	35	2
4	Proceedings Papers	65	1.731	2,347	2,535	23
5	Editorial Materials	28	0.745	265	275	9
6	Early Access	20	0.532	10	10	1
7	Book Chapters	18	0.479	1,363	1,366	11
8	Letters	14	0.373	269	272	5
9	Corrections	6	0.160	14	14	3
10	Retracted Publications	1	0.027	16	16	1
11	Retractions	1	0.027	1	1	1

TP, total publications; CAWS, citing articles without self-citations; TCAWS, times cited articles without self-citations.

### Data Analysis and Visualization

Developed by Professor Chaomei Chen, CiteSpace is a bibliometric and visual analysis tool specializing in exploring collaborations, priorities, internal structures, hotspots, and likely trends in a certain area (23). Therefore, we utilized CiteSpace (version 5.8) to perform collaboration network analysis (countries/regions, institutions, authors, and journals), co-citation analysis (authors, journals, and references), dual-map, citation burst detection for references. The specific parameters used in CiteSpace were set as follows: time slicing (from January 2001 to December 2021 years per slice=1), text processing (title, abstract, author keywords, and keywords plus), node type (one option chosen at a time from country, institution, author, keyword, source, co-cited journal, co-cited author, and co-cited reference), link strength (cosine), link scope (within slices), selection criteria (g-index, k=25 or k=20), pruning (Minimum Spanning Tree and Pruning Sliced Networks) and others followed the default. Betweenness centrality is an indicator to measure the importance of nodes in the network (in addition to degree centrality, closeness centrality, etc.). This indicator is used by CiteSpace to find and quantify the value of literature, and a purple circle is used to emphasize such literature (or authors, journals, institutions, etc.) (24).

Developed by Leiden University, VOSviewer does well in map creation, visualization, and exploration based on network data (25). VOSviewer (version 1.6.17) was used to create the keywords co-occurrence and cluster map based on text data. We used natural language processing algorithms to extract terms from the fields of titles and abstracts, supplemented with VOSviewer corpus files. We cleaned the data by combining “Mesenchymal Stem Cells”, “Mesenchymal Stromal Cells”, “BMSCs”, and “hMSCs” as “Mesenchymal Stem Cells” and excluding nominal terms such as “*in vivo*” and “model” in term analysis.

Microsoft Office Excel 2019 was used to manage the number of articles published in the year and used CiteSpace and VOSviewer software to analyze the distribution of countries/regions, institutions, authors and co-cited authors, journals, and co-cited authors journal, dual-map, cluster map, and keywords co-occurrence. Besides, we obtained the 2019 impact factor (IF) and JCR division of journals from the Web of Science.

## Results

### Temporal Distribution Map of Publications and Citations

**Figure 2:**
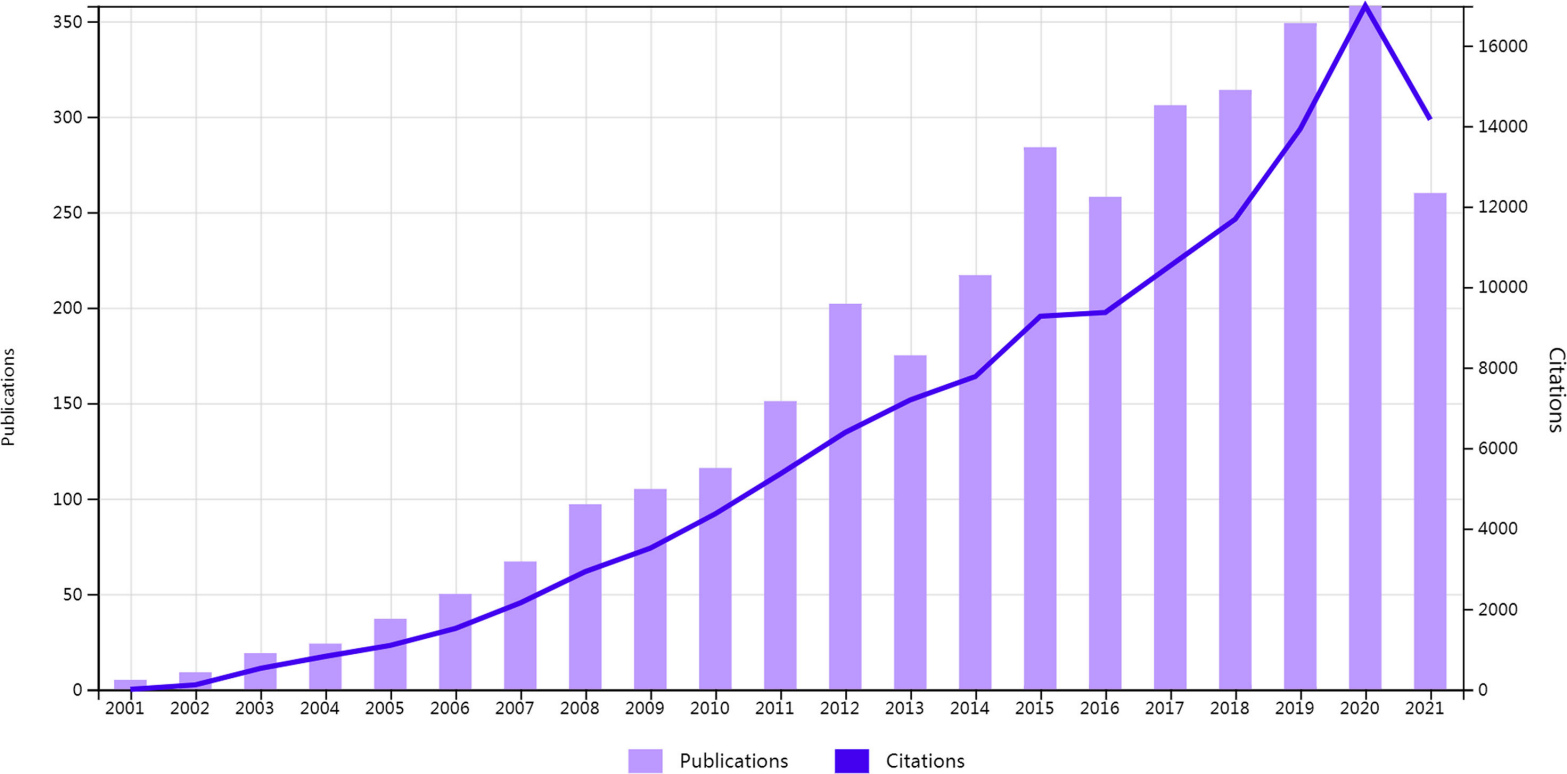
Temporal distribution map of publications and citations.

First, the change in annual publications and citation frequency reflects the speed and progress of this study and the degree of research focus in this subject (26). From 2001 to 2021, the annual publications on MSCs in liver diseases included 3,404 related articles, which showed an increasing trend (**Figure 2**). The number of papers published on this topic continuously increased from 2001 to 2012, with a modest drop in 2013, a rise from 2014 to 2015, and a drop in 2016. The number of articles has steadily increased between 2017 and 2020, exceeding 300 in 2017. Additionally, **Figure 2** also depicts the upward trend of citation frequency from 2001 to 2020.

### Spatial Distribution Map of Countries/Regions and Institutions

**Figure 3:**
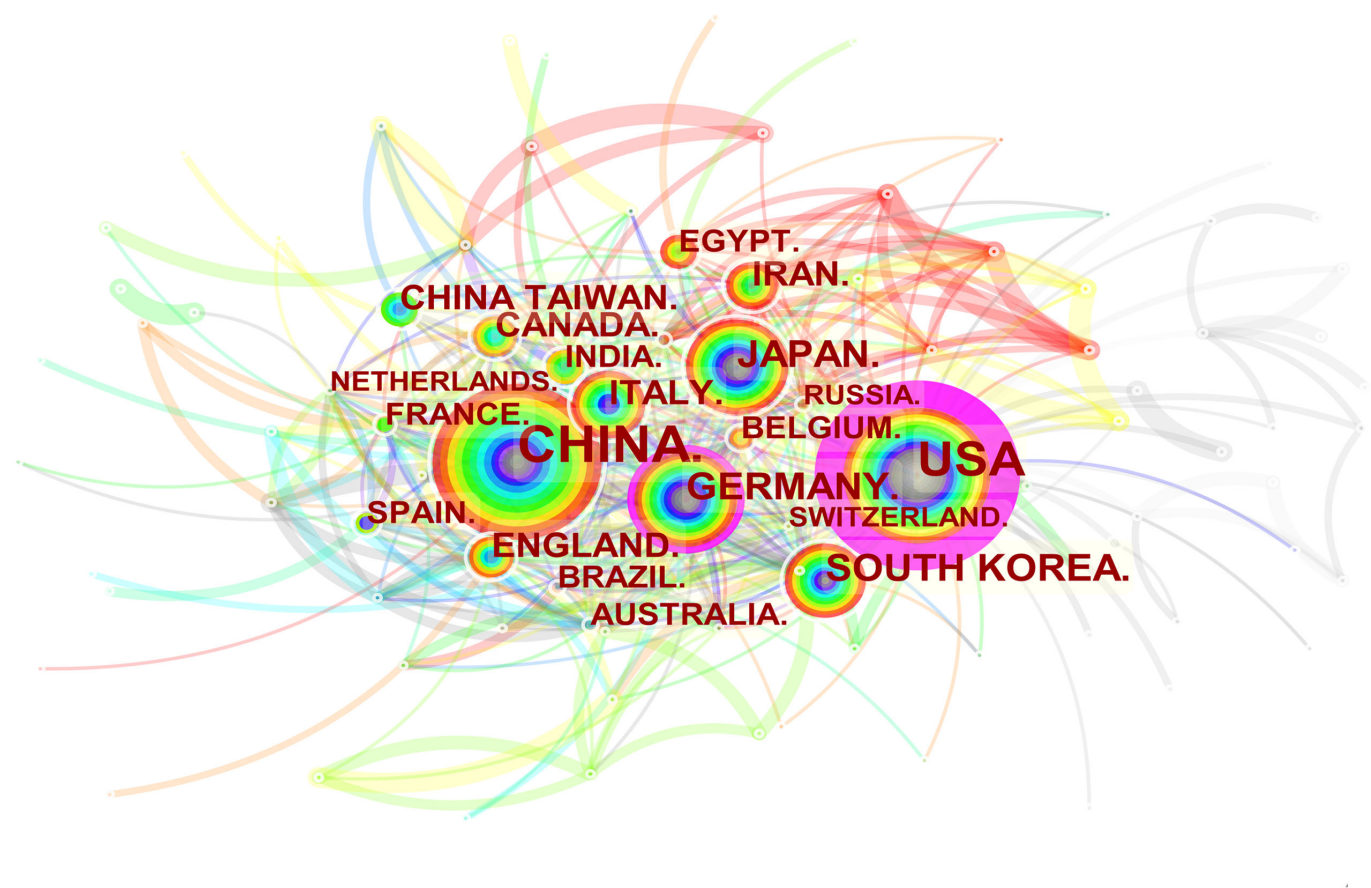
Spatial distribution map of countries/regions. The size of the node reflects the frequencies, and the links indicate the collaboration relationships. The color of the node and line represent different years. The outermost purple ring represents the centrality level, and the nodes with high centrality are considered to be the key points in the research field.

**Figure 4:**
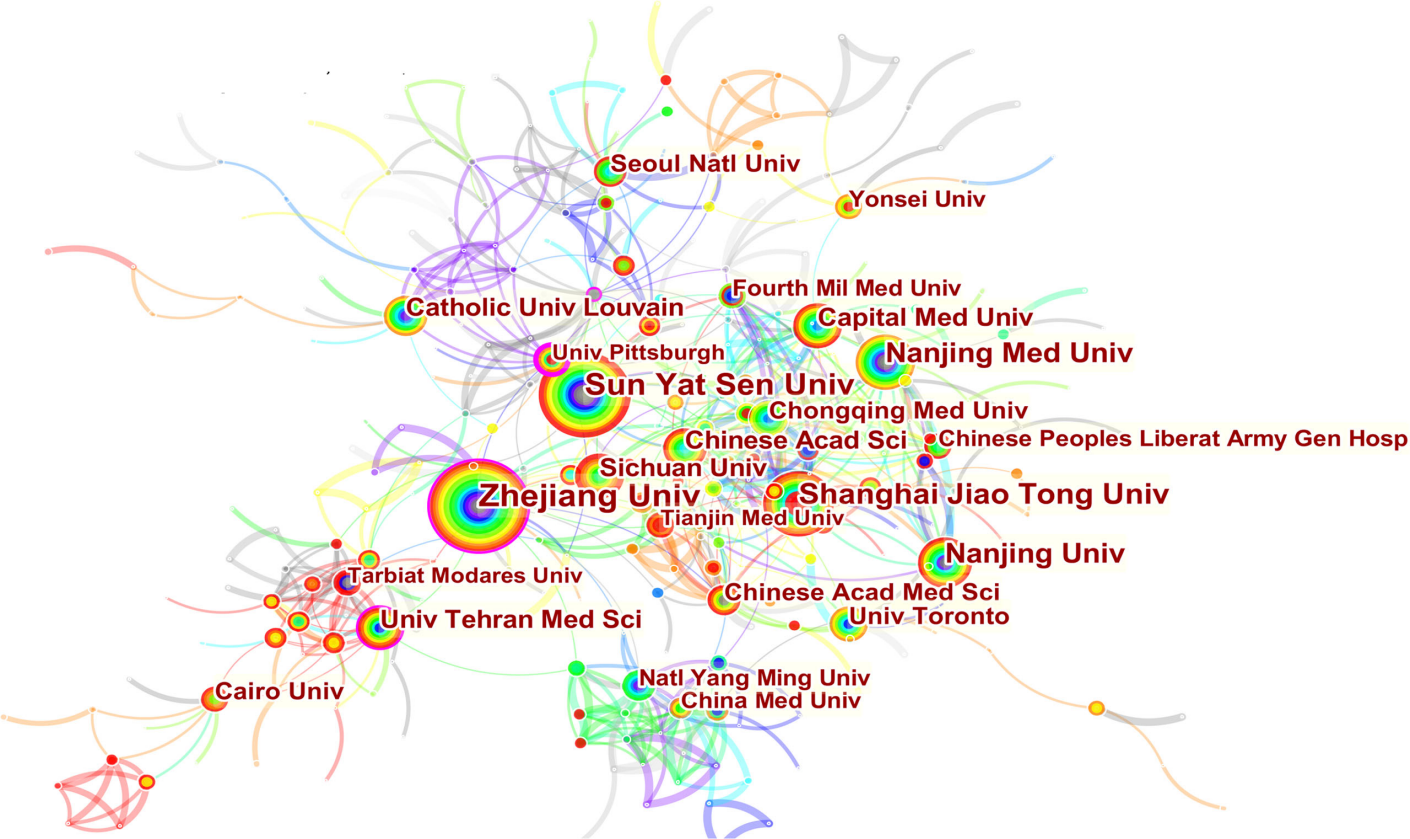
Spatial distribution map of institutions.

3251 institutions from 113 different countries/regions contributed the publications to MSC research in the field of liver diseases. We ranked 10 high-productivity countries/regions and institutions according to **Table 2**. China (1105/32.46%) and the USA (684/20.09%) published the most articles, which are more than five times higher than those of other countries, followed by Japan (227/6.70%), Germany (218/6.40%), and South Korea (213/6.26%). In addition, Zhejiang University (107, 3.4%) published the most papers, followed by Sun Yat-Sen University (77, 2.26%) and Shanghai Jiao Tong University (63, 1.85%). Among the top 10 productive institutions, China was the home to most institutions, excluding Tehran University of Medical Sciences in Iran and Catholic University Louvain in Belgium. Additionally, several countries and affiliations, such as the USA (0.54), Germany (0.20), England (0.11), China (0.11), University Pittsburgh (0.22), Zhejiang University (0.20), and Harvard University (0.13), showed high centrality, circled in purple in **Figures 3** and **4**. This finding suggests that the MSC study in these countries and institutions may have played a critical role in researching liver diseases. Each circle in the diagram represents a nation, with the size of the circle indicating the country’s publishing output. The lines that connect the circles represent international collaboration, and the broader the lines, the tighter the cooperation. There is active cooperation among countries and affiliations, such as Germany, Iran, Egypt, Italy, Belgium and England, Iraq and Indonesia, Tarbiat Modares University, Tianjin First Central Hospital, and Shahid Beheshti University. However, most nations and research institutions are scattered, lacking consistent and extensive cooperation.

**Table 2:** Publications in the 10 most productive countries/regions and institutions.

Rank	Country/Regions	Year	Count(%)	Centrality	Institutions	Year	Count(%)	Centrality
1	China	2004	1105(32.46)	0.11	Zhejiang Univ	2008	107(3.14)	0.2
2	USA	2001	684(20.09)	0.54	Sun Yat Sen Univ	2007	77(2.26)	0.06
3	Japan	2007	227(6.70)	0.07	Shanghai Jiao Tong Univ	2012	63(1.85)	0.04
4	Germany	2001	218(6.40)	0.2	Nanjing Med Univ	2007	58(1.70)	0.03
5	South Korea	2008	213(6.26)	0.01	Nanjing Univ	2008	55(1.61)	0.01
6	Italy	2008	145(4.26)	0.08	Capital Med Univ	2009	39(1.15)	0.06
7	Iran	2007	144(4.23)	0.02	Univ Tehran Med Sci	2007	39(1.15)	0.11
8	England	2002	119(3.50)	0.11	Sichuan Univ	2015	38(1.12)	0.01
9	Canada	2007	99(2.91)	0.04	Chinese Acad Sci	2008	37(2.91)	0.07
10	China Taiwan	2007	93(2.73)	0.01	Catholic Univ Louvain	2007	35(1.03)	0.04

### Visual Analysis of Authors and Co-Cited Authors

**Figure 5:**
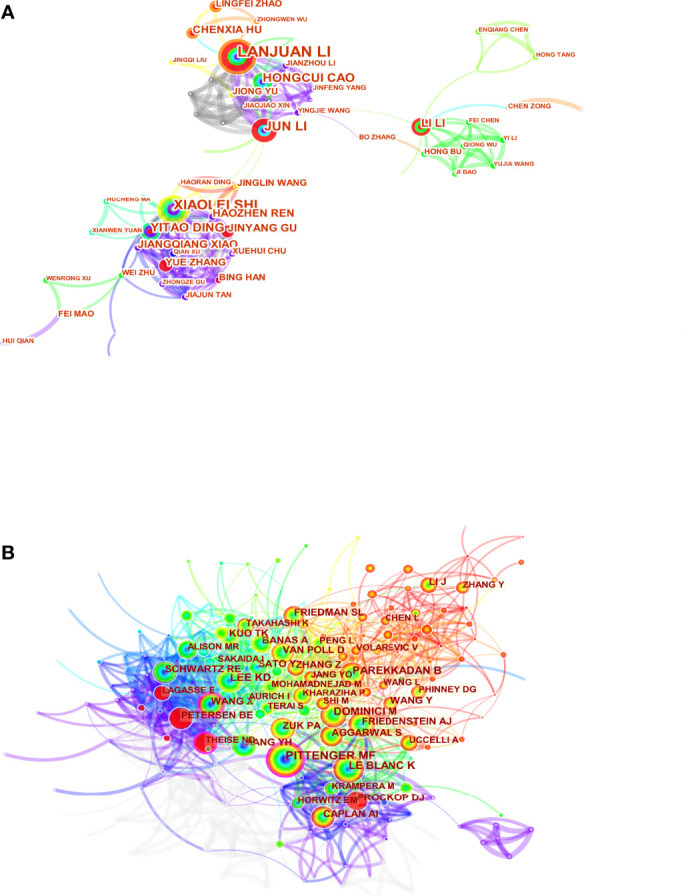
Visual analysis of authors **(A)** and co-cited authors **(B)**.

Highlighting the contributions of influential researchers, such as the authors of many co-occurring or co-cited papers in specific fields, could help academics along this path and provide further directions and guidelines (27). 18167 authors and 77521 co-cited authors were associated with MSCs in liver medicine. The top ten productive authors are listed in **Table 3**. Li L of Zhejiang University State Key Laboratory for Diagnosis and Treatment of Infectious Diseases tied for top place in this discipline with the most publications published (n=50), followed by Shi X (n=31), Li J (n=19), and Ding Y (n=16). It is worth noting that the betweenness centrality is relatively low (≤ 0.01), implying that the authors have little effect on each other’s work. The node size represents the number of studies published by the author, with larger nodes representing more published papers. The closer the collaboration between the two writers is, the shorter the distance between the two nodes. The purple nodes represent early published articles, while the red nodes represent recently published articles. Co-citation analysis is a significant part of bibliometrics. Co-cited authors refer to two or more authors cited by another or more papers simultaneously, constituting a co-cited relationship. Among the top 10 co-cited authors have been cited more than 200 times, Pittenger MF (551) was the most frequently co-cited author, followed by Dominici M (384), Le Blanc K (305), and Lee KD (258). We can see that Pittenger MF (0.17) and Parekkadan B (0.10) have high centralities. As **Figure 5** shows, there is a network of communication and cooperation among authors and co-cited authors in this research field.

**Table 3:** Top 10 authors and co-cited authors.

Rank	Author	Count (%)	Centrality	Co-cited author	Citations	Centrality
1	Lanjuan Li	31	0	Pittenger MF	551	0.17
2	Xiaolei shi	22	0.01	Dominici M	384	0.08
3	Jun li	19	0.01	Le Blanc K	305	0.08
4	Yitao Ding	16	0	Lee KD	258	0.04
5	Mustapha Najimi	15	0	Parekkadan B	255	0.1
6	Li Li	13	0.01	Wang Y	227	0.02
7	Hossein Baharvand	12	0	Caplan AI	222	0.09
8	Hongcui Cao	12	0	Banas A	217	0.05
9	Yang Yang	12	0	Friedenstein AJ	211	0.08
10	Jinyang Gu	11	0	Friedman SL	210	0.03

### Visual Analysis of Journals and Co-Cited Journals

To search for the most productive and influential journals, we use VOSviewer software to visualize published journals related to MSCs in livers diseases. The results showed that 3404 articles were published in 926 academic journals. As shown in **Table 4**, the Stem Cell Research & Therapy (126 publications, IF: 6.832) published the most articles concerning MSCs in liver diseases, followed by Plos One (81 publications, IF: 3.24) and Stem Cells International (75 publications, IF: 5.443). Among the top ten journals, four were in the Q1 JCR division, and five had an Impact Factor (IF) of more than five (**Table 4**). Through the analysis of the co-citation of periodicals, we can see the contribution of each periodical to this field. Among 8,941 co-cited journals, six journals had citations over 3,000. As presented in **Table 4**, hepatology had the most co-citations (citations: 6,307, IF: 17.425), followed by Stem cells, Blood, and Plos One. According to the 2020 Journal citation reports (JCR), 90% were at the Q1 JCR division except for Plos One. Six of the top ten co-cited journals had an IF of more than ten, and eight were from the United States.

**Table 4:** Document types of the publications.

Rank	Journal	Count(%)	JCR	IF(2020)	Co-cited journal	Citation	JCR	IF(2020)
1	Stem cell research & therapy	126	Q1	6.832	Hepatology	6307	Q1	17.425
2	Plos one	81	Q2	3.24	Stem cells	5632	Q1	6.227
3	Stem cells international	75	Q2	5.443	Blood	4232	Q1	22.113
4	Cell transplantation	65	Q2	4.064	Plos one	3826	Q2	3.24
5	Stem cells and development	57	Q2	3.272	Proceedings of the national academy of sciences of the United States of America	3575	Q1	9.58
6	Cytotherapy	56	Q1	5.414	Nature	3318	Q1	49.962
7	World journal of gastroenterology	55	Q2	5.742	Science	2850	Q1	47.728
8	International journal of molecular sciences	48	Q1	5.923	Biomaterials	2793	Q1	12.479
9	Biochemical and biophysical research communications	43	Q2	3.575	Journal of hepatology	2750	Q1	25.083
10	Stem cells	41	Q1	6.227	Transplantation	2644	Q1	4.939

**Figure 6:**
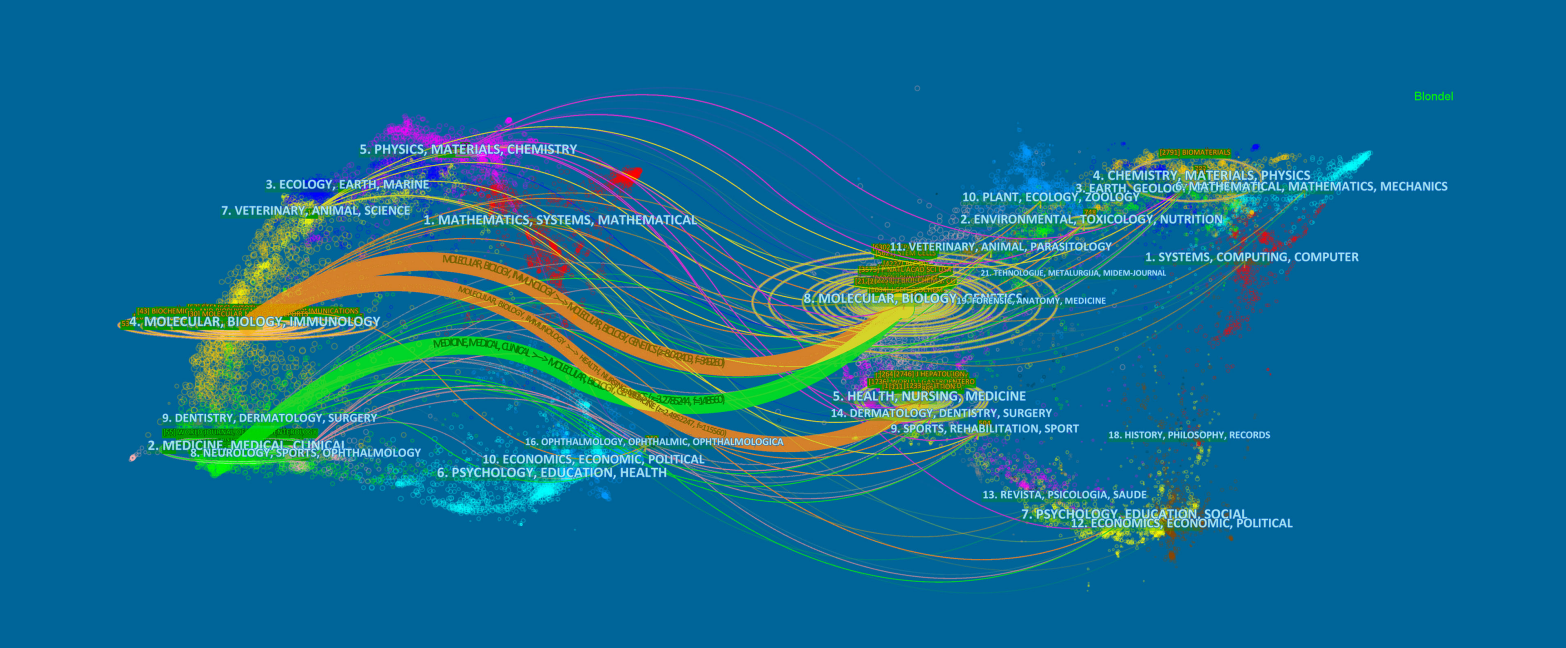
The dual-map overlay of journals. The citing journals were on the left, the cited journals were on the right, and the colored path represents the citation relationship.

Designed by Chen and Leydesdorff L, the analysis of dual-map overlays reveals patterns of a scientific portfolio respecting a global scientific literature map (28). The global base map depicts the interlinkages of over 10,000 scientific journals, which are further divided into regions representing subject-level publication and citation activity (29). **Figure 6** shows a dual-map overlay concerning MSCs and hepatopathy articles published between 2001 and 2021. All coloured curves originating from the citing (the left map) map and pointing to the cited (the right map) map represent the paths of the citation links. **Figure 6** indicates that the papers published in Molecular/Biology/Genetics and Healthy/Nursing/Medicine journals are often cited by the Molecular/Biology/Immunology journals, while Molecular/Biology/Genetics journals are often cited by Medicine/Medical/Clinical journals.

### Visual Analysis of Co-Citation and Clustering Network

Small and Marshakova presented co-citation analysis as a research tool to assess the link between articles in 1973, and it was then integrated into literature co-citation analysis (30, 31). When two or more articles appear in the reference list of one or more subsequent publications simultaneously, this is referred to as a co-cited relationship (29). The process of mining the co-citation relationship in a dataset is regarded as a co-citation analysis, and this analysis is used to measure the relationship between the two documents and visualize the co-occurrence of their references. The mapping co-citation analysis of literature is the core function of CiteSpace (32). It is also the first function to use and discuss the theory when CiteSpace is developed and used. Citing and cited articles are interrelated and continuously extended systems in which the knowledge structure of a research field can be fairly expressed (33). Therefore, the cross-reference of papers reflects the structure and dynamics of a knowledge domain (34). Citing articles and cited articles represent the research frontier and knowledge base (29). Moreover, the analysis of typical clusters can help us understand the knowledge structure and dynamic evolution of MSCs in liver diseases. 3404 citing articles and valid references have been analyzed to identify the homogeneous clusters of highly cited literature related to the studies on MSCs and liver disorders.

**Figure 7:**
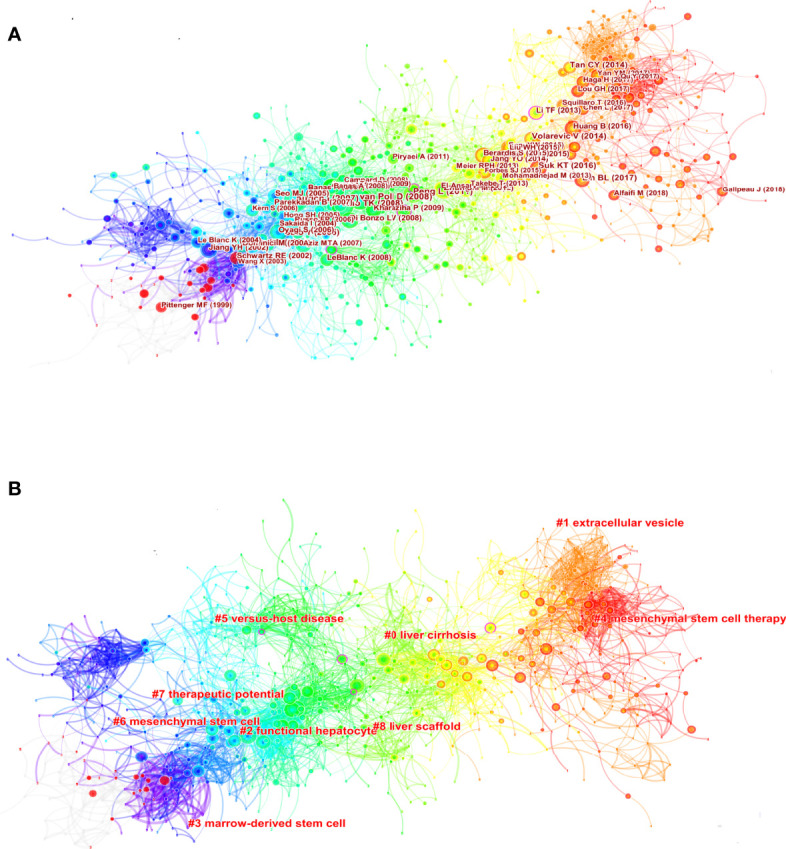
Visual analysis of co-citation **(A)** and clustering network **(B)**. The nodes in the figure represent the co-citation literature, and the links between nodes represent the co-citation relationship. Large nodes or nodes with red tree rings are either highly referenced or erupted. All cluster labels were extracted from titles of citing articles using the log-likelihood ratio algorithm.

**Figure 7A** displays co-citations of the 122490 references, the first author, and the year of the top 10 most cited references. Each circle represents a reference. The size of the circle is proportional to the citation frequency. The link between the two circles represents two references cited in the same article among the 3404 articles (citing articles) retrieved in this study. Similarly, line thickness is positively correlated with co-citation frequency. More information on the top 10 references cited is presented in **Table 5**. The most co-cited reference performed by Kuo TK et al. in 2008 was an original article published in Gastroenterology, entitled “Stem cell therapy for liver disease: parameters governing the success of using bone marrow mesenchymal stem cells” (35), followed by an article entitled “Autologous Bone Marrow Mesenchymal Stem Cell Transplantation in Liver Failure Patients Caused by Hepatitis B: Short-Term and Long-Term Outcomes” (36).

**Table 5:** Top 10 co-cited references.

Ranks	Title	Journal	Co-citation	Centrailty	Ref
1	Stem cell therapy for liver disease: Parameters governing the success of using bone marrow mesenchymal stem cells	Gastroenterology	79	0.06	35
2	Autologous Bone Marrow Mesenchymal Stem Cell Transplantation in Liver Failure Patients Caused by Hepatitis B: Short-Term and Long-Term Outcomes	Hepatology	72	0.09	36
3	Functional integration of hepatocytes derived from human mesenchymal stem cells into mouse livers	Gut	72	0.08	37
4	Mesenchymal stem cell-derived molecules directly modulate hepatocellular death and regeneration in vitro and in vivo	Hepatology	71	0.04	38
5	Transplantation With Autologous Bone Marrow-Derived Mesenchymal Stem Cells for Alcoholic Cirrhosis: Phase 2 Trial	Hepatology	69	0.06	39
6	In vitro hepatic differentiation of human mesenchymal stem cells	Hepatology	66	0.01	7
7	Human mesenchymal stem cells xenografted directly to rat liver are differentiated into human hepatocytes without fusion	Blood	60	0.01	40
8	Concise Review: Therapeutic Potential of Mesenchymal Stem Cells for the Treatment of Acute Liver Failure and Cirrhosis	Stem cells	59	0.05	41
9	Hepatocyte differentiation of mesenchymal stem cells from human adipose tissue in vitro promotes hepatic integration in vivo	Gut	58	0.02	8
10	Allogeneic Bone Marrow-Derived Mesenchymal Stromal Cells for Hepatitis B Virus-Related Acute-on-Chronic Liver Failure: A Randomized Controlled Trial	Hepatology	56	0.02	42

Cluster analysis can show the knowledge structure of the research field. Through the analysis of co-cited literature and cluster, we can summarize research topics in specific fields explore hotspots and research directions (43). Then, based on the co-citation state of 122,490 references to articles cited through CiteSpace, a hierarchical clustering network is generated if the two publications have many similar references and are often homogeneous. The largest nine clusters extracted from the references of the 3404 citing articles are shown in **Figure 7B**. Cluster labels are well-known noun phrases extracted from the title of citing articles using logarithmic likelihood ratio (LLR) algorithm, including #0 liver cirrhosis, #1 extracellular vesicle, #2 functional hepatocyte, #3 marrow-derived stem cell, #4 mesenchymal stem cell therapy, #5 versus-host disease, #6 mesenchymal stem cell, #7 therapeutic potential, #8 liver scaffold (**Figure 7B**). The total Q-value was 0.7246, and each cluster had a weight mean silhouette of 0.8906 or higher, suggesting that the cluster quality was reasonable. The number of cluster tags is inversely related to the number of articles per cluster included. Purple nodes represent early clustering labels that included #3 marrow-derived stem cell and #6 mesenchymal stem cell, while red nodes represent recent clustering labels such as #5 mesenchymal stem cell therapy and #1extracellular vesicle.

**Figure 8:**
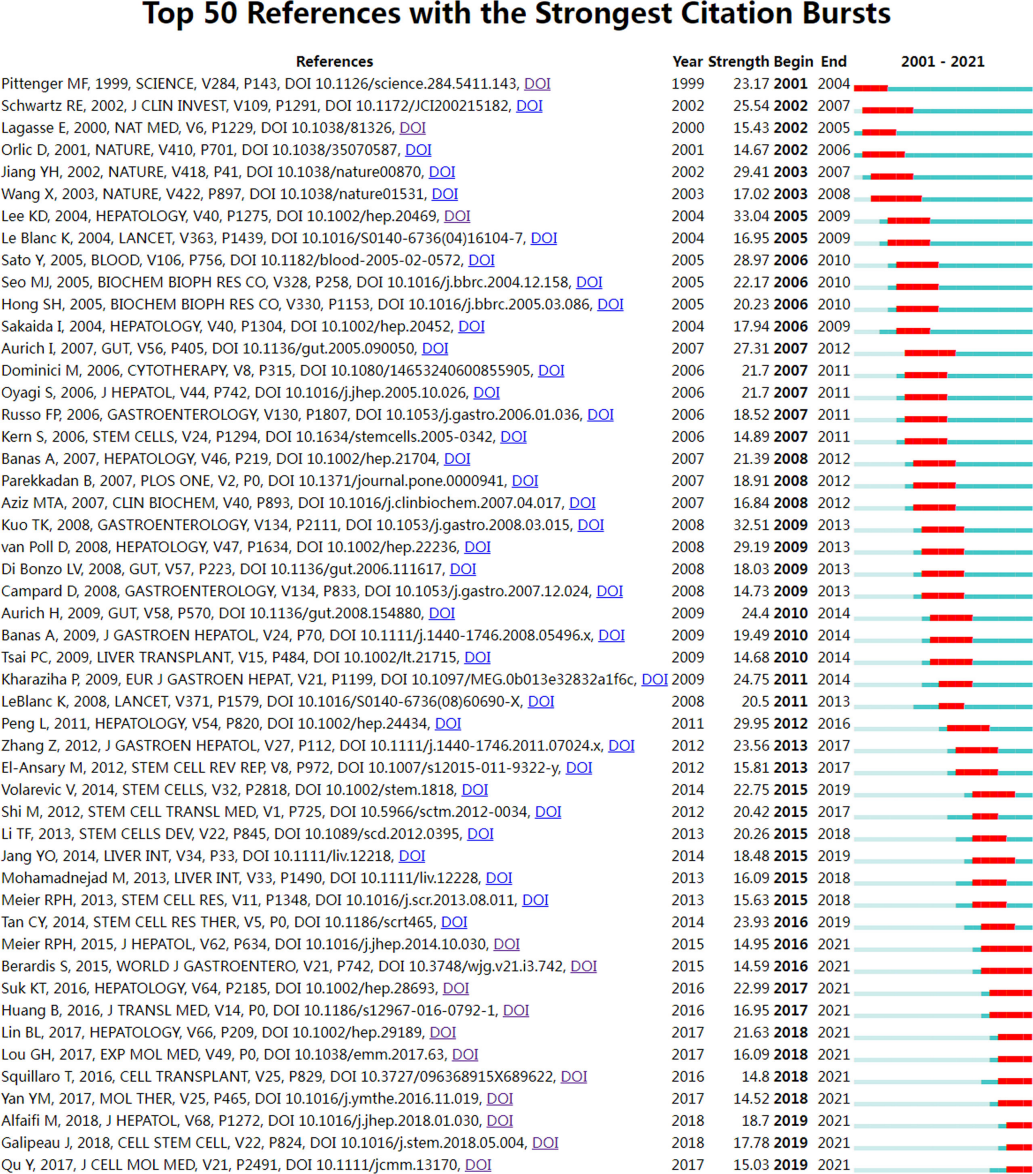
Visual analysis of references bursts. The intensity value reflects the cited frequency. The red bar indicates citation frequency; green bars indicate fewer citations.

References burst are defined as those frequently cited for some time (44). We set the burst duration to at least two years in CiteSpace, from which we detected 50 of the most bursty references (**Figure 8**). **Figure 8** shows that the first co-citation burst began in 2001, entitled “Multilineage Potential of Adult Human Mesenchymal Stem Cells” (45). Notably, eleven references (22%) were in burstiness until 2021, which implies that the research related to MSCs in liver diseases research may continue to explode in the future. The paper with the strongest burstiness (strength=33.04) was entitled “*In vitro* hepatic differentiation of human mesenchymal stem cells”, published in Hepatology by Kuan-Der Lee *et al.* in 2004, with citation bursts from 2005 to 2009 (7).

### Visual Analysis of Keyword Co-Occurrence

**Figure 9:**
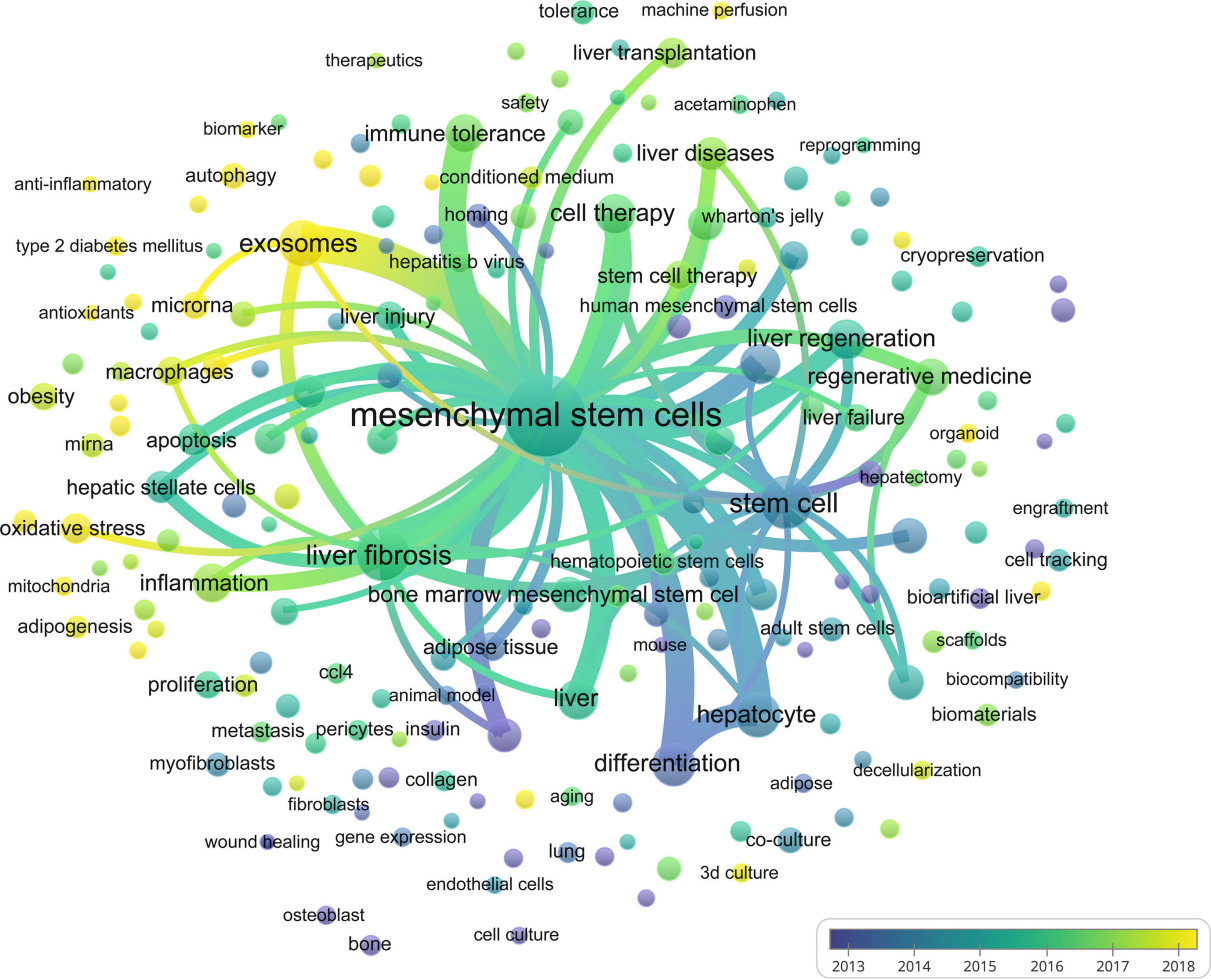
Visual analysis of keyword co-occurrence. Different colors of the circles indicated the average year of the studies according to the bar on the lower right corner.

Keywords are standardized terms selected from titles and texts to represent the paper’s theme and make it easier to archive information (46). When investigating the knowledge structure of science, keywords may be used to accurately identify research frontiers and hot areas, which is a useful approach to bibliometric analysis. Apart from search terms, author keywords extracted from the titles and abstracts of 3,404 papers were analyzed by VOSviewer. A total of 60902 keywords were extracted, of which 2110 keywords appeared more than ten times, and 402 keywords appeared more than fifty times. As we can see from **Figure 9** and **Table 6**, mesenchymal stem cells were the most important term with 987 co-occurrences, followed by liver fibrosis, stem cell, exosomes, hepatocyte, and differentiation. In the keywords co-occurrence visualization diagram, author keywords are marked with different colors according to their average publication years. “differentiation”, “cell transplantation”, “hepatocyte”, and “stem cell” were mainly found in the early years (**Figure 9**). Keywords such as “exosomes”, “oxidative stress”, “organoid”, “autophagy”, and others are highlighted in yellow, suggesting that these domains have grown in popularity in recent years and may become a hot topic in the prospective. However, different from the co-citation analysis, the keyword co-occurrence analysis has found some new terms, such as “machine perfusion”, “liver transplantation”, and “microRNAs”, which also may become research hotspots in the future.

**Table 6:** The top 20 keywords.

Rank	Keyword	Occurrences	Total link strength	Rank	Keyword	Occurrences	Total link strength
1	mesenchymal stem cells	987	916	11	inflammation	91	83
2	liver fibrosis	288	267	12	hepatocyte	86	85
3	stem cell	244	219	13	regenerative medicine	80	76
4	exosomes	179	166	14	acute liver failure	74	70
5	differentiation	137	126	15	cell transplantation	69	66
6	liver	113	111	16	tissue engineering	68	67
7	cell therapy	109	104	17	hepatocytes	64	60
8	liver regeneration	107	96	18	liver diseases	64	64
9	transplantation	103	93	19	bone marrow	61	61
10	immune tolerance	98	91	20	apoptosis	56	54

## Discussion

This study is the first bibliometric analysis of the structural and temporal dynamics of MSCs in liver diseases. According to the WoSCC database, as of October 2, 2021, a total of 18167 authors from 3251 institutions in 113 countries have published 3404 papers on MSCs and hepatopathy in 926 academic journals. We used CiteSpace and VOSviewer to evaluate the spatial and temporal distribution, authors’ contributions, and journals of 3404 retrieved articles. We used literature co-citation and keyword co-occurrence analysis to identify the research knowledge base, hotspots, and frontiers in each period and define the theme’s core evolutionary route. Then, we identified the current research frontiers of MSCs in the field of liver diseases.

The absence of research on MSCs in liver disorders before 2001 implies a dearth of relevant data. A tiny number of studies began to appear between 2001 and 2006, and research was still in its infancy at that time. During this period, Jiang Y found that MSCs could differentiate into visceral mesoderm, including liver cells *in vitro* (47), which resulted in developing a novel technique for the long-term investigation of liver regenerative medicine. More and more researchers began to pay attention to the role of MSC therapy in liver diseases (47). Research publications on MSCs have exploded in the last ten years. Clinical data showed that autologous MSC infusion was utilized to treat end-stage liver disease with good tolerance (48). Hundreds of clinical trials are already underway, and thousands of individuals have received MSC treatment (49–52), indicating that MSC research in the field of liver disease science is expected to become a hot spot and research direction in the future.

Concerning the spatial distribution map of countries/regions and institutions, **Table 2** and **Figures 3**, **4** showed that China, the USA, and Japan were the top 3 high-yield nations. Furthermore, the United States and Germany were the earliest countries to take up the MSC study, followed by the Netherlands, Japan, France, England, and Sweden; these four countries are among the ten most productive. Professor Freeman (Freeman, L, 1978/1979), an American sociologist, proposed betweenness centrality as an index that measures the extent to which a point lies in the “middle” of other “point pairs” in the graph (53). It is mainly used to measure the bridge function value of nodes in the whole network structure (54). Of the top 10 countries in **Table 2**, the United States has the highest betweenness centrality (0.54), which plays a key bridge role in national cooperation networks worldwide. Interestingly, the number of publications in China is substantially more than that in the United States, but the betweenness centrality of the former was lower than that of the latter. This result might be explained because China alone publishes a huge quantity of papers, resulting in low betweenness centrality. The top 10 institutions were from three countries. It is worth noting that, despite its late start (2008), Zhejiang University was able to become the center of the highest number of publications (107) and influence (0.2) in a short time. Furthermore, we discovered extensive collaboration between the University of Pittsburgh, Harvard University, the Chinese Academy of Medical Sciences, and other institutions, signifying significant contributions to MSCs in liver illnesses.

To identify the most productive authors, we rank authors based on the total number of articles they published on MSCs related to liver diseases and evaluate them in combination with other indexes to offer a more comprehensive view of the most prolific author (55, 56). Li L (50 papers) is the author with the highest number of publications, followed by Shi X (31 papers), Li J (19 papers), and Ding Y (16 papers). It is noteworthy that the betweenness centrality is relatively low (≤ 0.01), implying a lack of communication and collaboration among authors in this work. It can be seen from **Figure 5A** that the distribution of researchers is relatively scattered, and the main researchers, such as Lanjuan Li, Yitao Ding, and Li Li, do not form a network, indicating a lack of academic exchanges between researchers. Therefore, it is strongly advised that academics from the United States, China, and other countries break down academic boundaries, collaborate, and communicate to advance MSC research and development in liver illnesses. Three writers were co-cited more than 300 times among the total cited authors, with Pittenger MF receiving the most co-citations (384 citations), followed by Dominici M (384 citations) Le Blanc K (305 citations).Furthermore, we can see that Pittenger MF (0.17) and Parekkadan B (0.10) have high centralities. In 1999, Pittenger MF and his colleagues identified the multilineage potential of MSCs *in vitro* (45). This finding reveals that hMSCs can proliferate widely and maintain the ability to differentiate multiple various cell types *in vitro*, establishing their stem cell properties, and their cultivation and selective differentiation should provide a further understanding of the important precursors of this diverse type of tissue. This research will confirm that MSCs have the potential to provide innovative therapies for injured or diseased tissue, as well as the theoretical underpinning for MSCs to differentiate into liver cells. Moreover, Parkkadan B and his colleagues further found that systemic infusion of MSC conditioned medium (MSC-CM) induces liver protection in acute liver injury by inhibiting cell death and stimulating repair procedures (38, 57). This discovery aided in the paradigm change from basic MSCs to extracellular vesicles (EVs). These prominent academic pioneers and emerging young researchers can attract a large number of talented researchers to join MSCs in the clinical transformation of liver diseases.

Journal and co-cited journal analysis can provide considerable information, which helps researchers choose appropriate journals to submit papers (58). Our study found that nearly one-fifth (19.01%) of the total papers published in the top ten most active journals related to MSCs in the field of hepatopathy, suggesting that the literature distribution is relatively concentrated. **Table 4** showed that Stem Cell Research & Therapy (126 publications, IF: 6.832) published the most articles, while Hepatology attracted the largest number of co-citations. Both of these are journals on medicine research, experimental medicine, cell biology, cell & tissue engineering, which is consistent with the dual-map analysis (**Figure 6**). The dual-map overlay of journals represents a subject distribution of academic journals (59, 60). **Figure 6** showed two main citation paths from Molecular/Biology/Genetics co-cited journals to Medicine/Medical/Clinical journals and from Molecular/Biology/Genetics and Healthy/Nursing/Medicine co-cited journals to Molecular/Biology/Immunology journals, implying that MSCs related studies in liver disease have developed from cell biology to clinical medicine (61). Meanwhile, journals with IF>5 accounted for most of the top 10 journals (60%) and co-cited journals (80%), suggesting that these journals have interests and play significant roles in this field, which reminds scholars interested in this topic should pay more attention to these journals.

Marrow-derived stem cells could differentiate into nonhematopoietic cells of multiple tissues, including epithelial cells of the liver, gastrointestinal (GI) tract, and myocytes of heart and skeletal muscle (62). It can be seen from **Figure 7B** that it is the earliest Cluster # 3 (marrow-derived stem cell), which was the first report that bone marrow-derived stem cells had hepatic differentiation potential (63–65). Subsequently, Schwartz, R.E and his colleagues found that postpartum bone marrow-derived MSCs combined with a variety of growth factors, cytokines, and compounds (i.e., HGF, epidermal growth factor [EGF], fibroblast growth factor [FGF]-2/-4, etc.) could increase the expression of hepatocyte markers such as HNF-3b, GATA4, transthyretin, albumin, a-fetoprotein, CK18, and CK19 (66). In addition, Cluster # 2 (functional hepatocyte) further studied the hepatogenic differentiation capacity of MSCs in many independent studies on BM-MSC (37, 40, 67), hAT-MSCs (8), UCB-MSC (68) and human placenta-MSCs (hPMSCs) (69). In this cluster, MSCs can effectively rescue experimental liver failure, promote liver regeneration, and provide a potential alternative therapy for liver transplantation (35). The aforementioned two clusters aided the advancement of liver tissue engineering (Cluster # 8) and provided a theoretical foundation for using MSCs in the treatment of a variety of liver illnesses (41). Due to the low retention rate and low survival rate, MSCs need to be combined with a variety of biologically active tissue structures to effectively integrate into the target tissue, such as polyethene glycol hydrogels (70), alginate (71), collagen (72, 73) and chitosan (74). MSCs could be transformed *in vitro* into functional hepatocyte-like cells through induction using a specific biomatrix scaffold-decellularized liver matrix (75). The natural ECM framework allows for specified differentiation of stem cells into mature cell types in space, making this an appealing 3D bioscaffold for cell biology and tissue regeneration investigations.

In addition to the properties of hepatic differentiation, Cluster # 6 (mesenchymal stem cell) demonstrates that MSCs have low immunogenicity and an extensive immunoregulatory role *via* cell contact-dependent mechanisms and soluble factors (76). MSCs regulate many types of innate immune cells in inflammatory sites, including macrophages, dendritic cells, and natural killer (NK) cells (77–79). One of the main adaptive immunomodulation for MSCs is the regulation of T cells. MSCs not only inhibit the proliferation of T cells (80) but also inhibit the reaction of naive T cells and memory antigen-specific T cells (81). MSCs also inhibit the activation of cytotoxic CD8^+^ T cells and the differentiation of Th1 and Th17 cells (82) while promoting the Treg differentiation and activity (83, 84). In addition, the role of MSCs on the activation, proliferation, and antibody production of B cells has received less attention in liver diseases, which poses new requirements and challenges for researchers (85).

Their immunoregulatory capabilities and differentiation capacity give them Cluster#7 (therapeutic potential) for clinical treatment of pathological liver disorders in which inflammation and immunological, pathological responses play a key role. These liver diseases mainly include Cluster#0 (liver cirrhosis) and Cluster#5 (graft-versus-host disease). In Cluster#7 and Cluster#5, we found that MSCs were the clinical, theoretical basis for treating liver diseases. Cluster#0 (liver cirrhosis) is the largest cluster from **Figure 7**. The main focus of this cluster is to explore the role of MSCs in the randomized controlled trials (RCT) studies of liver cirrhosis from the perspective of clinical application (39, 42, 51, 86).

In basic studies, the mechanism of MSCs in treating liver cirrhosis has been evaluated from multiple perspectives. Chronic injury factors such as hepatitis virus and alcohol can gradually destroy the endothelial barrier and liver cells, causing inflammatory cell infiltration and activating hepatic stellate cells (HSCs) (87). Activation of HSCs (aHSCs) obtains a phenotype of myofibroblasts characterized by increased expression of α-smooth muscle actin (α-SMA) and increased production of ECM components, growth factors and cytokines, which is the main determinant during liver fibrosis (88). In the progression of liver fibrosis caused by different chronic injury factors, MSCs migrate to the liver fibrosis microenvironment to participate in liver injury repair. MSCs have a direct anti-fibrosis effect on HSCs by MSC-secreted cytokines/growth factors or cell-cell contact, which can inhibit the activation of HSCs and the potential of producing ECM and induce apoptosis of HSCs. MSCs have an anti-fibrotic impact on HSCs through a paracrine mechanism, such as matrix metalloproteinases-13(MMP-13), MMP-9 (89), MFGE8 (10, 90), tumour necrosis factor-inducible gene 6 protein (TSG-6) (91, 92) and hepatocyte growth factor (HGF) (93). Transplantation of MSCs enhanced the activity of MMP-9 and MMP-13 and attenuated activation of tissue inhibitors of metalloproteinase1 (TIMP-1), resulting in increased degradation of ECM proteins in the fibrotic liver (89, 94). MFGE8 secreted by MSCs downregulates the expression of TGFβ1 receptor by binding to α_v_β_3_ integrin on HSCs and interferes with the activation of TGFβ1 mediated HSCs, thereby promoting fibrosis regression (10). TSG-6 induces HSCs transformation into stem cell-like cells *in vitro*, and TSG-6-treated HSC-derived organoids can repair fibrotic liver (92). HGF derived from MSCs can also accelerate HSCs apoptosis, and MSCs cultured with HGF can improve serum albumin level, reducing liver fibrosis (93). In addition, MSCs inhibit HSCs activation and proliferation through direct cell-cell contact mode. Chen *et al.* found that MSCs induced arrest of HSCs in G0 cells *via* Notch-dependent pathway and significantly inhibited the proliferation and α-SMA expression of HSCs (95). MSCs also have an indirect anti-fibrosis impact by regulating immune cells, such as macrophages, and decreasing the activity of HSCs (96). In addition, MSCs conditioned medium and extracellular vesicles have been shown to reduce liver fibrosis (97) greatly. Although some literature suggests that the potential contribution of bone marrow-derived cells, such as fibrocytes and MSCs, to fibrogenic myofibroblasts has not been excluded (98, 99), some clinical trials using autologous and allogeneic MSCs transplantation have been carried out in patients with fibrosis, with a slight improvement of clinical parameters (such as albumin, creatinine) without serious adverse reactions, which indicates that MSCs have potential therapeutic effects on liver diseases (50, 100). However, few clinical trials involving MSCs improving liver failure and survival rates following liver transplantation, posing new challenges for researchers.

Actually, several classes of pharmacological agents are being used for the treatment of liver fibrosis, dependent on the aetiology, staging and progress of liver fibrosis (101, 102). Although the role of MSCs in mitigating fibrosis is encouraging, concomitant pharmacological agents remain a challenge for the treatment of liver fibrosis. Melatonin (MT) is the product of the pineal gland and has a variety of physiological functions. This hormone is an effective targeted therapy for liver fibrosis. Excitedly, Mias C*. et al.* found high expression of MT receptors (MT1and MT2) in MSCs, and it could enhance survival (103) and homing (104) of MSCs through a receptor-mediated mechanism. Preconditioning of MSCs with MT showed lower expressions of TGF-β1 and Bax and lowered ALT content but higher expressions of MMPs and Bcl2 with the MSCs group in the treatment of liver fibrosis (105). Similarly, vitamin E (Vit E) is widely considered one of the strongest antioxidants in nature, Vit E pretreated MSCs reduced the Timps/Mmps and promoted the degradation of ECM protein by inhibiting TGF-β1 signaling pathway during liver fibrosis (106). Most medications for liver fibrosis promote MSCs, but some may limit their viability or function. For example, rapamycin inhibits hepatic fibrogenesis by regulating the balance of Th17/Treg cells (107), but the beneficial role of MSCs will be antagonized by rapamycin *via* decreasing cell viability, differentiation, and proliferation (108). Therefore, more consideration should be given when selecting the combination therapy with MSCs.

Although Cluster#5 (mesenchymal stem cell therapy) has been reported to treat liver diseases in laboratory, preclinical and clinical trials, some issues still need attention. Long-term liver fibrosis can promote the abnormal proliferation, regeneration and repair of hepatocytes, making hepatocytes prone to spontaneous mutations, thus developing into hepatocellular carcinoma (HCC). In this process, MSCs, as a part of the fibrosis inflammatory microenvironment matrix, may be involved in the initiation and progression of HCC (109, 110). In the initial stage of HCC (Is), MSCs showed protective effects against drug damage by reducing DNA damage and ROS accumulation. In addition, MSCs in Is also have anti-inflammatory and anti-liver fibrosis effects. However, in the progressive stage of HCC (Ps), MSCs promote HCC formation not only by promoting cancer cell proliferation but also by promoting stem cell-like characteristics and epithelial-mesenchymal transition (EMT) of hepatoma carcinoma cell (111). Furthermore, when MSCs differentiated into hepatocytes after malignant transformation, the abnormal expression or localization of some genes may be related to tumour phenotype, such as β-catenin (112). Gleeson, B. M. *et al.* demonstrated that tissue factor (TF) is expressed on the surface of MSCs, which is the key promoter of the soluble coagulation cascade. This expression profile *in vitro* leads to the prethrombotic phenotype of MSCs and aggravates the complications of acute myocardial infarction (AMI), including microvascular obstruction (113). The potential tumorigenicity and microvascular obstruction of MSCs *in vivo* inhibit the clinical application of MSCs in the current regenerative medicine. Moreover, the short life span, easy agglomeration, and low transplantation rate of MSCs also limit the clinical application (114). With the further evolution of clusters, MSC-derived Cluster#1 (extracellular vesicles) in liver diseases gradually emerge.

With the further evolution of clusters, MSC-derived Cluster#1 (extracellular vesicles) in liver diseases gradually emerge. Recently, evidence has suggested that MSCs exerted their therapeutic effects in a paracrine manner Parekkadan B found that MSCs-CM derived from MSCs can reverse fulminant hepatic failure (57). MSCs conditioned medium (MSCs-CM) includes free soluble factors and EVs, which are divided into three categories, including exosomes (30–100 nm in diameter), microvesicles (100–1000 nm in diameter), and apoptotic bodies (500–2000 nm in diameter), may promote tissue regeneration, immune regulation, and angiogenesis by mediating intercellular micro-communication and transporting paracrine factors (115–117). Exosomes contain various lipids, proteins, and nucleic acids, such as lncRNAs and miRNAs, which play an important role in regulating homeostasis (118). MSCs-derived exosomes without self-replication have a lower risk of ectopic differentiation, tumorigenesis, immune rejection, and genetic instability (119). Meanwhile, Tamura *et al.* demonstrated that MSCs-derived exosomes pass through microvascular and are broadly distributed in damaged liver tissues (120). MSCs-derived exosomes improve liver function and hepatocyte proliferation and reduce apoptosis and liver necrosis in multiple liver disease models (97, 121, 122). Exosomes derived from MSCs have attracted much attention in regenerative medicine and tissue engineering. There is a scarcity of large-scale clinical evidence to back this up, and future research is needed to overcome these constraints.

Co-citation determines the topic’s knowledge base and clustering evolution, and keyword co-occurrence analysis can be the research hotspot and frontier of the topic. Excitedly, keyword co-occurrence analysis is a further extension and expansion of hot spots, some new terms, such as “machine perfusion”, “liver transplantation”, and “microRNAs”, which also may become research hotspots in the future. The term “machine perfusion” refers primarily to the continuous perfusion of transplants to mimic the body’s physiological state. Transplants can recover metabolism and even function during perfusion, especially at room temperature with matrix and oxygen supply in the machine perfusion system (123). As is well known, liver transplantation is still the definitive treatment for patients with end-stage liver failure, but the number of acceptable donor organs limits this definitive treatment. The emergence and application of (donation after circulatory death) DCD provide the possibility to solve the shortage of clinical liver supply (124). However, the long-term hepatic ischemia-reperfusion injury (IRI) and intracellular reactive oxygen species (ROS) injury lead to the damage of DCD donor liver structure and function, which may lead to complications such as primary non-function after liver transplantation (125). Machine perfusion has become a novel strategy for liver graft preservation, and the treatment with MSCs has unique advantages in the clinical application during perfusion in recent years (126, 127). Yang *et al.* confirmed that MSCs could inhibit macrophages activation, reduce ICAM expression, improve epithelial cell injury, and improve liver microcirculation of DCD during normothermic machine perfusion (128, 129). In addition, several new organ preservation technologies such as ischemic pre-conditioning (IPC), ischemic post-conditioning (IPostC) and remote ischemic conditioning (RIC) can also improve the availability of transplanted liver (130). The clinical application of new technologies combined with MSCs in liver transplantation will undoubtedly be another research hotspot.

MicroRNAs, as a unique class of non-coding RNAs, regulate the expression of their target genes at post-transcriptional level (131). Many studies have shown that microRNAs derived from exosomes of MSCs hepatocytes play a crucial role in the pathological and physiological processes of the liver, including hepatocyte differentiation, diseases and HCC. Cui, L. *et al.* found that miR-1246, miR-1290, miR-30a, miR-148a, miR-424 and miR-542-5p were highly expressed in MSCs during liver differentiation (132). Ectopic overexpression of the seven microRNAs can stimulate MSCs to transform into functional hepatocytes, improving liver injury induced by CCL4. Inhibition of let-7b caused upregulation of liver-enriched transcription factors, an increase in the expression of miR-122, and accumulation of MSCs in the G0/G1 phase of the cell cycle, activating hepatic differentiation (133). In the pathological and physiological processes of the liver, MSCs decreased TNFRSF21 (DR6) expression and hepatocyte apoptosis and improved ACLF by inducing miR-20a-5p expression in exosomes and hepatocytes (134). Exosomal miRNA-299-3p inhibited the inflammatory response and NLRP3 activation in the transplantation models to repair liver tissue (135). Interestingly, a variety of microRNAs in the extracellular vesicles of MSCs show anti-hepatoma effects, such as miR−199a−3p, miR-199a, miR-15a and miR-375 (136–139). In addition, long non-coding RNAs (lncRNA) and circular RNAs (circRNA), as components of non-coding RNAs, also play an irreplaceable role in the underlying mechanism of MSCs in the treatment of liver diseases (140, 141).

Through the analysis and visualization of bibliometrics, this field’s structural and temporal dynamics can be understood to some extent. The research has some certain limitations inherent in bibliometrics. First, the data is retrieved only from the WoSCC database, and some important findings published in other databases may be missed. However, WoSCC is an authoritative and comprehensive database in the medical field. WoSCC data represent most of the information to some degree (142–144). Secondly, data are included only in research articles and reviews. Nevertheless, the amount of data we analyze is large enough to reflect MSCs research in liver diseases. Lastly, VOSviewer and CtieSpace may miss some information because they cannot analyze the full text of publications, leading to bias as reported in other bibliometric studies.

## Conclusion

MSCs are generally considered a safe and potentially relevant treatment strategy for liver diseases. The bibliometric analysis provides an objective and quantitative method for evaluating trends and frontiers of MSCs in liver diseases. With the help of CiteSpace and VOSviewer, we have a deeper understanding of the research status, evolution path, frontier hotspots, and future trends of MSCs in liver diseases in recent 20 years. The leading countries are China and the US; however, it is necessary to strengthen cooperation and exchanges among countries, institutions, and authors. Increasing numbers of articles published in international core journals show a significant impact. Tissue engineering, translational medicine, and extracellular vesicle of MSCs in liver diseases will focus on future research. This study can provide important clues for researchers to understand this field’s structural and temporal dynamics.

## Author Contributions

BS, conception and design, data analysis and interpretation, and manuscript writing. Y-FQ, S-HR and Q-FP, collection and assembly of data, data analysis, and interpretation. HQ, Z-BW, H-DW, G-ML, Y-LZ, C-LS, J-YZ, and XL, collection of data and data analysis. HW, conception and design, financial support, administrative support, manuscript writing, and final approval of the manuscript. All the authors have read and approved the final content of this manuscript.

## Funding

This work was supported by grants to HW from the National Natural Science Foundation of China (No. 82071802), Science and Technology Project of Tianjin Health Commission (No. TJWJ2021MS004), Tianjin Key Medical Discipline (Specialty) Construction Project and to Z-BW from the National Training Program of Innovation and Entrepreneurship for Undergraduates (No. BK11020220).

## Conflict of Interest

The authors declare that the research was conducted in the absence of any commercial or financial relationships that could be construed as a potential conflict of interest.

## Publisher’s Note

All claims expressed in this article are solely those of the authors and do not necessarily represent those of their affiliated organizations, or those of the publisher, the editors and the reviewers. Any product that may be evaluated in this article, or claim that may be made by its manufacturer, is not guaranteed or endorsed by the publisher.
